# Piperine Induces Hepatic Low-Density Lipoprotein Receptor Expression through Proteolytic Activation of Sterol Regulatory Element-Binding Proteins

**DOI:** 10.1371/journal.pone.0139799

**Published:** 2015-10-02

**Authors:** Ayasa Ochiai, Shingo Miyata, Makoto Shimizu, Jun Inoue, Ryuichiro Sato

**Affiliations:** Department of Applied Biological Chemistry, Graduate School of Agricultural and Life Sciences, The University of Tokyo, Tokyo, Japan; Hokkaido University, JAPAN

## Abstract

Elevated plasma low-density lipoprotein (LDL) cholesterol is considered as a risk factor for atherosclerosis. Because the hepatic LDL receptor (LDLR) uptakes plasma lipoproteins and lowers plasma LDL cholesterol, the activation of LDLR is a promising drug target for atherosclerosis. In the present study, we identified the naturally occurring alkaloid piperine, as an inducer of LDLR gene expression by screening the effectors of human LDLR promoter. The treatment of HepG2 cells with piperine increased LDLR expression at mRNA and protein levels and stimulated LDL uptake. Subsequent luciferase reporter gene assays revealed that the mutation of sterol regulatory element-binding protein (SREBP)-binding element abolished the piperine-mediated induction of LDLR promoter activity. Further, piperine treatments increased mRNA levels of several SREBP targets and mature forms of SREBPs. However, the piperine-mediated induction of the mature forms of SREBPs was not observed in SRD–15 cells, which lack insulin-induced gene–1 (Insig–1) and Insig–2. Finally, the knockdown of SREBPs completely abolished the piperine-meditated induction of LDLR gene expression in HepG2 cells, indicating that piperine stimulates the proteolytic activation of SREBP and subsequent induction of LDLR expression and activity.

## Introduction

High-plasma low-density lipoprotein (LDL) cholesterol levels are a major risk factor for atherosclerosis and coronary heart disease [1]. The liver is a major organ that uptakes LDL particles via LDL receptor (LDLR)-meditated endocytosis and has the ability to metabolize up to 70% of plasma LDL [2]. Thus, increased LDLR expression in the liver ameliorates atherogenic lipid profiles and suppresses plasma LDL-cholesterol levels.

LDLR expression is regulated by transcription, mRNA degradation, post-translational modification, and protein degradation [3–6]. Accordingly, the widely used cholesterol-lowering statins inhibit HMG-CoA reductase, which performs the rate-limiting step for cholesterol biosynthesis, and stimulate LDLR gene expression, and statin-mediated LDLR gene expression follows the activation of sterol regulatory element-binding protein–2 (SREBP–2) [7].

Transcription factors of the SREBP family, including SREBP-1a, SREBP-1c, and SREBP–2, are central to transcriptional control of genes related to cholesterol and fatty acid metabolism [8]. SREBP–2 preferentially regulates the expression of genes involved in cholesterol metabolism, including LDLR, whereas SREBP-1c regulates genes involved in the fatty acid biosynthetic pathway. SREBP-1a has been shown to regulate the genes involved in cholesterol and fatty acid biosynthesis [9–11]. All three SREBPs are synthesized as membrane proteins of the endoplasmic reticulum (ER) and are processed to liberate N-terminal halves that function as transcription factors in the nucleus. This proteolytic activation is tightly regulated by interactions with ER membrane proteins, SREBP cleavage-activating protein (SCAP), and insulin-induced gene (Insig). The depletion of cellular sterols leads to the dissociation of the SCAP/SREBP complex from Insig, and the subsequent binding of SCAP to the COPII proteins Sar1 and Sec23/24 leads to the incorporation of the SCAP/SREBP complex into COPII-coated vesicles. Consequently, SREBPs enter the Golgi and are processed by site–1 and -2 proteases [9].

Piperine is a pungent constituent of black pepper (*Piper nigrum*) and long pepper (*Piper longum*) and is classified as an alkaloid [12]. As a pharmacological agent, piperine has demonstrated anti-cancer, anti-inflammatory, and anti-microbial activities [13,14]. In addition, several reports indicate that oral administration or dietary piperine improves high-fat diet-induced metabolic abnormalities, such as dyslipidemia, hepatic steatosis, and insulin resistance [15–18]. Although piperine reduces serum cholesterol levels in diet-induced obese mice [15–17], the mechanism underlying this effect remains unknown.

In the present study, we performed luciferase reporter assays using the LDLR promoter region and identified food components that stimulate LDLR gene promoter activity. These experiments showed that piperine stimulates LDLR expression and LDL uptake into HepG2 cells. Further experiments showed that piperine stimulates maturation of SREBPs by accelerating SREBP translocation from the ER to the Golgi. Accordingly, piperine-mediated LDLR gene expression was abolished in SREBP-knockdown HepG2 cells.

## Materials and Methods

### Reagents

Piperine, 25-hydroxycholesterol (25-HC), fluvastatin, and lipoprotein-deficient serum (LPDS) were purchased from Sigma. The details of commercial suppliers of other screened compounds are available upon request. DiI-labeled LDL was obtained from Molecular Probes.

### Cell culture

Huh–7, HEK293, and HepG2 cells were obtained from ATCC. SRD–15 cells were kindly provided by Debose-Boyd RA (University of Texas Southwestern Medical Center) [19]. Huh–7/FAS-Luc cells (Huh–7 cells stably expressing a luciferase reporter driven by the LDLR promoter) were maintained in medium A (DMEM supplemented with 10% FBS, 100 U/ml penicillin, and 100 μg/ml streptomycin) containing 2 μg/ml blasticidin S. HepG2 and HEK293 cells were maintained in medium A. CHO–7 Chinese hamster ovary cells were grown in LPDS medium, and SRD–15 (CHO–7) cells lacking Insig–1 and -2 were maintained in medium B (DMEM/Ham’s F–12 supplemented with 100 U/ml penicillin, 100 μg/ml streptomycin, and 10% FBS). All cell cultures were incubated at 37°C under a 5% CO_2_ atmosphere.

### Plasmid construct

Reporter plasmids pLDLR-Luc, which contains the human LDLR promoter region from −595 to +36, and pLDLR-Luc-mSRE, which contains a mutant SREBP-binding element (SRE) sequence were described previously [20].

### Stable transfection of Huh–7 cells with the LDLR promoter-containing luciferase reporter plasmid

Huh–7 cells were plated in 60-mm dishes at a density of 8 × 10^5^ cells/dish and were cultured in medium A for 20 h. Cells were subsequently transfected with 7.62 μg of pLDLR-Luc (LDLR promoter) and 0.38 μg of pMAM2-BSD (blasticidin S deaminase) using lipofectamine 2000 according to the manufacturer’s instructions. Twenty-four hours after transfection, cells were plated in 100-mm dishes and were cultured in medium A containing 8 μg/ml blasticidin S. The medium was changed every second day until well-defined colonies were observed. Colonies were isolated using cloning cylinders in the presence of 2 μg/ml blasticidin S. The resulting stable cell lines were cultured in medium A or medium C (DMEM supplemented with 100 U/ml penicillin, 100 μg/ml streptomycin, and 12.5 μM of the HMG-CoA reductase inhibitor fluvastatin, and 50 μM sodium mevalonate containing 5% LPDS) under normal or sterol-depleted conditions for 16 h, respectively. The Huh–7/LDLR-Luc cell line was selected according to the induction of luciferase activity under cholesterol-depleted conditions.

### Luciferase assays in Huh–7/LDLR-Luc cells

Huh–7/LDLR-Luc cells were plated on 12-well plates at a density of 1 × 10^5^ cells/well, were cultured in medium A for 20 h, and were then incubated for 24 h in the absence or presence of test compounds at 100 μM. Luciferase activity and protein contents in cell extracts were measured as described previously [21]. Luciferase activities were normalized to total protein contents of cell extracts.

### Luciferase assays

Luciferase assays were performed as described previously [22].

### LDL uptake assays

LDL uptake assays were performed as described previously [23].

### Real-time PCR

Total RNA was extracted from HepG2 cells using Isogen (Wako) according to the manufacturer’s instructions, and cDNA was synthesized and amplified from 2 μg of total RNA using a high-capacity cDNA reverse transcription kit (Applied Biosystems). Quantitative real-time PCR (TaqMan probe and SYBR green) analyses were performed using an Applied Biosystems StepOnePlus instrument. Expressions of SREBP1 (TaqMan ID, Hs00231674_m1) and SREBP2 (Hs00190237_m1) were normalized to those of the glyceraldehyde-3-phosphate dehydrogenase (GAPDH; 4352934) control. Forward and reverse primer sequences were as follows: LDLR, 5′-CAGAGGCAGAGCCTGAGTCA–3′ and 5′-CGGGTGTCTCAGGCACTTAA–3′; HMG-CoA reductase, 5′-CTTGTGTGTCCTTGGTATTAGAGCTT–3′ and 5′-GCTGAGCTGCCAAATTGGA–3′; HMG-CoA synthase, 5′-GACTTGTGCATTCAAACATAGCAA–3′ and 5′-GCTGTAGCAGGGAGTCTTGGTACT–3′; squalene synthase (SQS), 5′-ATGACCATCAGTGTGGAAAAGAAG–3′ and 5′-CCGCCAGTCTGGTTGGTAA–3′; proprotein convertase subtilisin/kexin (PCSK9), 5′-ATCCACGCTTCCCTGCTGC–3′ and 5′-CACGGTCACCTGCTCCTG–3′; fatty acid synthase (FAS), 5′-GCAAATTCCGACCTTTCTCCAGAA–3′ and 5′-GTAGGACCCCGTGGAATGTC–3′; acetyl-CoA carboxylase 1 (ACC1), 5′-TGGGCCTCAAGAGGATTTGT–3′ and 5′-TCCACTGTTGGCTGATACATAGATG–3′; SREBP-1a, 5′-TCAGCGAGGCGGCTTTGGAGCAG–3′ and 5′-CATGTCTTCGATGTCGGTCAG–3′; and SREBP-1c, 5′-GGAGGGGTAGGGCCAACGGCCT–3′ and 5′-CATGTCTTCGAAAGTGCAATCC–3′, respectively.

### siRNA experiments

SREBP–1 (sc–36557) and SREBP–2 (sc–36559) siRNAs were obtained from Santa Cruz, and control siRNA (pGL2 luciferase) was obtained from Bonac. HepG2 cells were transfected with siRNA (40 pmol per six-well plate) using lipofectamine RNAiMAX (Invitrogen) according to the manufacturer’s instructions. The expression of SREBPs–1 and -2 was simultaneously suppressed using 20 pmol of each corresponding siRNA.

### Antibodies

Monoclonal anti-LDLR antibody (C7) was obtained from Millipore, and monoclonal anti-SREBP–1 (2A4) and anti-SREBP–2 (1C6) antibodies were obtained from Santa Cruz. Monoclonal anti-β-actin (AC–15) antibody was purchased from Sigma, and the anti-SREBP–2 polyclonal antibody (Rs004) was used as described previously [21]. Peroxidase-conjugated affinity-purified goat anti-rabbit and goat anti-mouse IgGs were purchased from Jackson Immunoresearch Laboratories.

### Immunoblotting

Cells were lysed in RIPA buffer containing 50 mM Tris-HCl (pH 8.0), 150 mM sodium chloride, 0.1% (w/v) SDS, 0.5% (w/v) deoxycholate, 1% (v/v) Triton X–100, and protease inhibitors. Lysates were subjected to SDS-PAGE, and proteins were transferred onto a PVDF membrane and probed with the indicated antibodies. Immunoreactive proteins were visualized using ECL (GE Healthcare) or Immobilon (Millipore) Western blotting detection reagents. Signals on membranes were detected and quantified using ImageQuant LAS 4000 mini (GE Healthcare).

### Statistical analysis

All data are presented as mean ± standard error of the mean (SEM). Statistical analyses were performed using Ekuseru-Toukei Ver.2.0 (Social Survey Research Information). Pairwise comparisons of treatments were made using Student’s *t* test, and multiple comparisons were made using one-way ANOVA followed by the Bonferroni test. Differences were considered significant when *P* < 0.05.

## Results

### Piperine increases the expression and activity of LDLR

**Fig 1:**
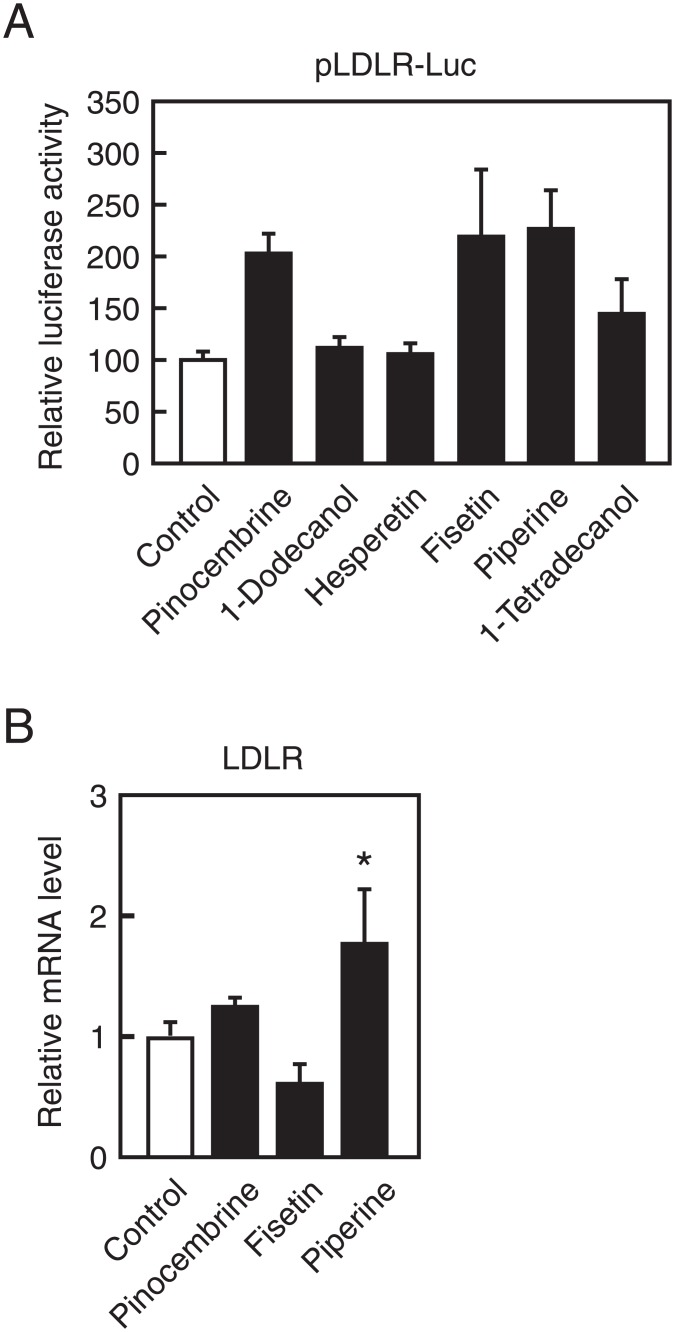
Effects of piperine on LDLR promoter activity and expression. (**A**) HEK293 cells were transfected with 50 ng of pLDLR-Luc and 50 ng of pEF-β-Gal. Cells were cultured in medium A for 24 h and were then treated with the indicated compounds (100 μM) for 24 h. Luciferase assays were performed as described in the Materials and Methods section. Promoter activity of pLDLR-Luc in the presence of the vehicle (DMSO) is represented as 100%. (**B**) HepG2 cells were cultured with the indicated compounds (100 μM) for 24 h, and total RNA was isolated. Real-time PCR analysis was performed, and mRNA expression levels were normalized to those of GAPDH and are presented relative to those in vehicle (DMSO)-treated controls. All data are expressed as mean ± SEM, *n* = 3; *P < 0.05.

**Fig 2:**
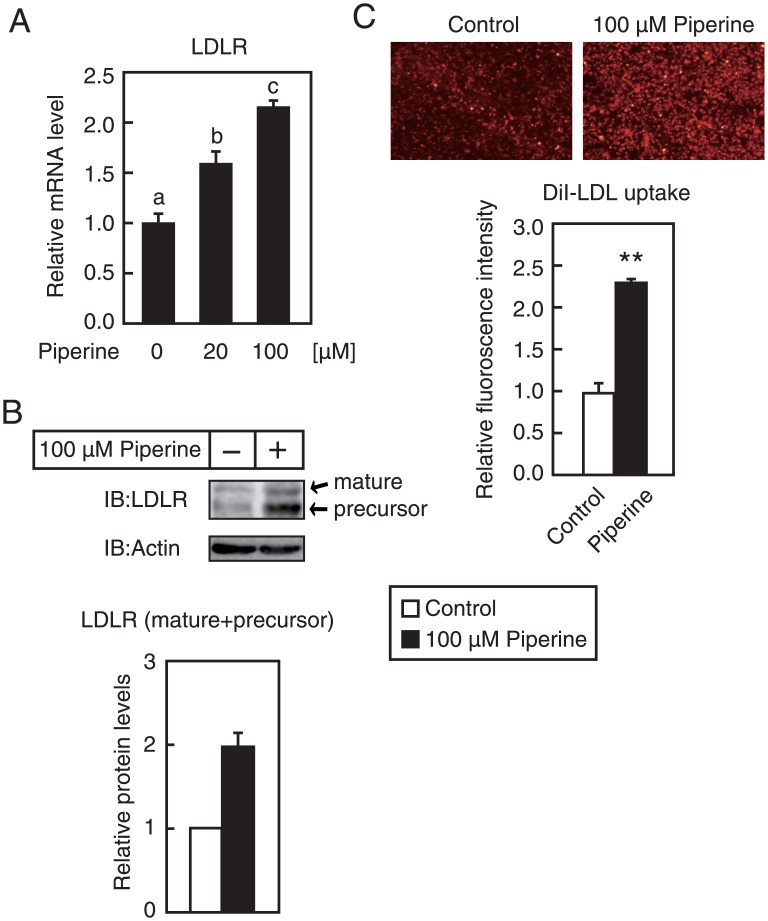
Piperine increases the expression and activity of LDLR. (**A** and **B**) HepG2 cells were cultured with the indicated concentration of piperine for 24 h, and total RNA and whole cell extracts were isolated. (**A**) Real-time PCR analysis was performed and mRNA levels were normalized to those of GAPDH mRNA and expressed relative to those in vehicle (DMSO)-treated controls. All data are expressed as mean ± SEM, *n* = 3. Differing letters indicate significant differences, *P < 0.05. (**B**) Whole-cell extracts were subjected to SDS/PAGE and immunoblotting (IB) with anti-LDLR or anti-β-actin antibodies (upper panel). Similar results were obtained in three separate experiments. The signals (n = 3) were quantified, and the signals of the control group are represented as 1 (lower panel). (**C**) HepG2 cells were cultured with piperine (100 μM) for 24 h and were then cultured in medium supplemented with 10 μg/ml DiI-labeled LDL for the last 5 h. The cells were then examined using fluorescence microscopy (upper panel), and relative fluorescence levels were normalized to total cellular protein contents (lower panel). All data are expressed as mean ± SEM, *n* = 3; *P < 0.01.

To identify compounds that increase the expression of LDLR, we established a stable cell line that expressed a luciferase reporter gene under the control of the LDLR promoter region from −595 to +36. Human hepatoma Huh–7 cells were transfected with pLDLR-Luc and the expression plasmid for blasticidin S deaminase and were then cultured in the presence of blasticidin S. Blasticidin S-resistant clones (Huh–7/LDLR-Luc cells) were isolated and expanded and were treated with approximately 100 food components at 100 μM for 12 h. Subsequent luciferase assays showed increased LDLR promoter activity in the presence of several food components (S1 Table), and those that increased luciferase activity by more than 1.5-fold were further examined in transient luciferase assays of LDLR gene promoter activity. As shown in Fig 1A, pinocembrine, fisetin, and piperine stimulated LDLR promoter activity by more than 1.5-fold when compared with a vehicle control. Among these, piperine treatments led to increased endogenous LDLR mRNA levels in HepG2 cells after 24 h (Fig 1B), and the effect was dose dependent (Fig 2A). LDLR protein is synthesized as a precursor form with an apparent molecular mass of 120 kDa (precursor in Fig 2B) and is then post-translationally modified to the mature form with an apparent molecular mass of 160 kDa (mature in Fig 2B). Treatment with piperine for 24 h increased the presence of the mature and precursor LDLR (Fig 2B). Thus, to determine whether treatment with piperine causes increased LDL uptake, HepG2 cells were treated with piperine for 24 h and were then incubated with the fluorescent-labeled LDL DiI-LDL for 5 h. Subsequent fluorescence microscopy showed increased DiI-LDL internalization by HepG2 cells in the presence of piperine (Fig 2C), suggesting that piperine-mediated increase of LDLR mRNA leads to increased LDL uptake.

### Piperine stimulates LDLR promoter activity via the SREBP-binding element

**Fig 3:**
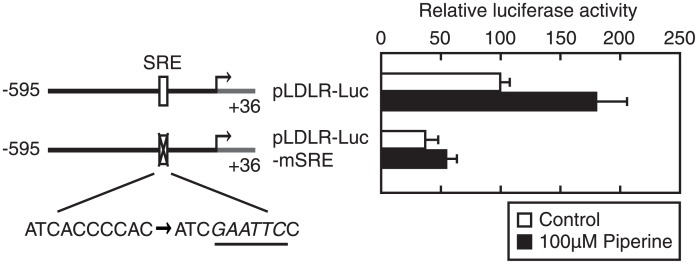
Piperine activates the LDLR promoter through the SREBP-binding element. HepG2 cells were transfected with 100 ng of the indicated reporter constructs and 100 ng of pEF-β-Gal. Cells were then cultured in medium A for 24 h and were treated with piperine (100 μM) for 24 h. Luciferase assays were performed as described in the Materials and Methods section, and promoter activity of pLDLR-Luc was calculated relative to that in the presence of the vehicle (DMSO). All data are expressed as mean ± SEM, *n* = 3; **P < 0.01.

SREBPs reportedly stimulate promoter activity of the LDLR gene [24]. Thus, to investigate the roles of SREBP in piperine-mediated stimulation of LDLR gene expression, reporter assays were performed using mutant LDLR promoters (Fig 3). In these experiments, the mutation of SRE resulted in inhibition of piperine responsiveness and decreased basal LDLR promoter activity. These results indicate that piperine stimulates LDLR promoter activity through SRE.

### Piperine activates SREBPs by stimulating proteolytic processing of SREBPs

**Fig 4:**
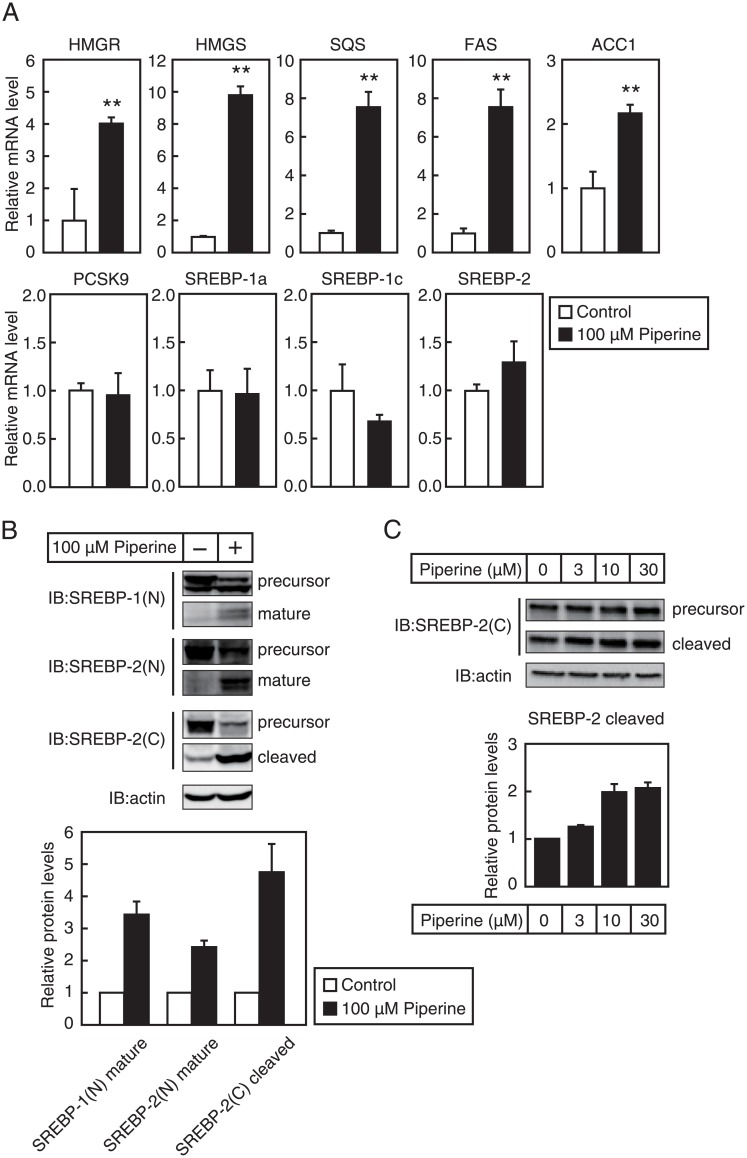
Piperine stimulates SREBP activity. (**A**) HepG2 cells were cultured with piperine (100 μM) for 24 h, and total RNA was isolated. Real-time PCR analyses were performed, and mRNA expression of target genes was normalized to that of GAPDH mRNA and was expressed relative to mRNA levels in vehicle (DMSO)-treated controls. All data are expressed as mean ± SEM, n = 3; **P < 0.01. (**B**) HepG2 cells were cultured with piperine (100 μM) for 3 h and whole-cell extracts were isolated for SDS/PAGE and IB with anti-SREBP–1 (2A4), anti-SREBP–2 (Rs004), anti-SREBP–2 (1C6), or anti-β-actin antibodies (upper panel). Similar results were obtained in three separate experiments. The signals (n = 3) were quantified, and the signals of the control group are represented as 1 (lower panel). (**C**) HepG2 cells were cultured with indicated concentrations of piperine for 24 h, and whole-cell extracts were isolated for SDS/PAGE and IB with anti-SREBP–2 (1C6) or anti-β-actin antibodies (upper panel). Similar results were obtained in three separate experiments. The signals (n = 3) were quantified, and the signals of the control group are represented as 1 (lower panel).

To determine whether piperine elevates endogenous mRNA levels of SREBP target, HepG2 cells were treated with piperine for 24 h, and quantitative real-time PCR analyses were performed (Fig 4A). In these experiments, piperine treatment caused a significant increase in the mRNA levels of HMG-CoA reductase, HMG-CoA synthase, SQS, FAS, and ACC1 in HepG2 cells, suggesting that piperine stimulates SREBP activity. However, piperine did not increase expression of the SREBP target gene PCSK9 or SREBP genes (Fig 4A). Thus, to investigate the effect of piperine on SREBP processing, HepG2 cells were treated with piperine for 3 h, and lysates were subjected to immunoblotting using anti-SREBP–1 and anti-SREBP–2 antibodies. SREBP–1 was detected by an antibody recognizing its N-terminus [SREBP–1(N)], whereas SREBP–2 was detected by antibodies recognizing its N- [SREBP–2(N)] and C- [SREBP–2(C)] termini. SREBP processing was stimulated by 3-hour treatments with 100 μM piperine, as indicated by increased mature and cleaved forms and decreased precursor forms (Fig 4B). Although 3 μM piperine only increased the presence of the cleaved form of SREBP–2 slightly, 10 and 30 μM piperine treatments led to marked increases (Fig 4C).

### Piperine stimulates SREBP processing under cholesterol-supplemented conditions

**Fig 5:**
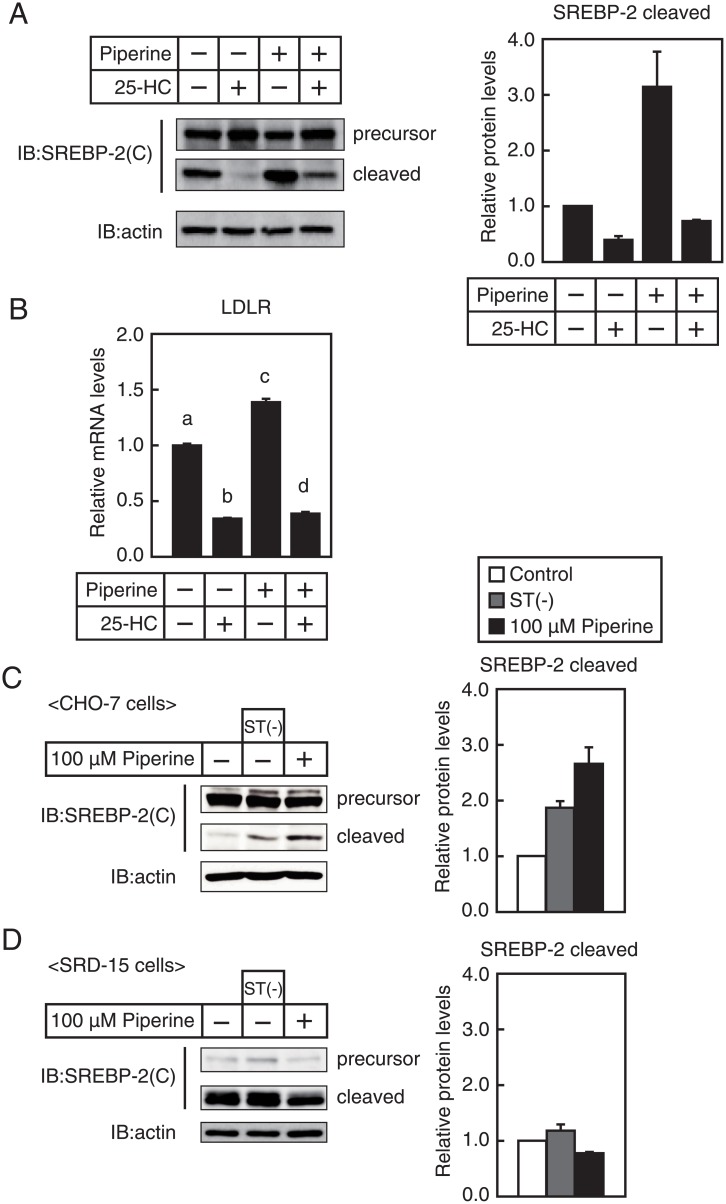
Effect of piperine on SREBP processing. (**A** and **B**) After preincubation with 1 μg/ml 25-HC for 30 min, HepG2 cells were cultured with piperine (100 μM) for 4 h, and whole-cell extracts and total RNA were isolated. (**A**) Whole-cell extracts were subjected to SDS/PAGE and IB with anti-SREBP–2 (1C6) or anti-β-actin antibodies (left panel). Similar results were obtained in three separate experiments. The signals (n = 3) were quantified, and the signals of the control group are represented as 1 (right panel). (**B**) Real-time PCR analysis was performed and mRNA levels were normalized to those of GAPDH mRNA and expressed relative to those in vehicle (DMSO)-treated controls. All data are expressed as mean ± SEM, *n* = 3. Differing letters indicate significant differences, *P < 0.05. (**C** and **D**) CHO–7 (**C**) and SRD–15 (**D**) cells were cultured with piperine (100 μM) or were depleted of sterols by incubation in medium C for 4 h, and whole-cell extracts were isolated for SDS/PAGE and IB with anti-SREBP–2 (1C6) or anti-β-actin antibodies (left panel). Similar results were obtained in three separate experiments. The signals (n = 3) were quantified, and the signals of the control group are represented as 1 (right panel).

SREBP processing is suppressed by sterols, which inhibit translocation of SCAP/SREBP complex from the ER to the Golgi. Thus, experiments were performed to determine whether piperine stimulates SREBP processing in the presence of sterols (Fig 5A). Piperine-mediated increases in the cleaved form of SREBP–2 were diminished but still observed in the presence of 25-HC (Fig 5A). Consistent with these results, piperine treatment caused a significant increase in the mRNA levels of LDLR regardless of the presence of 25-HC in HepG2 cells (Fig 5B).

### Piperine does not stimulate SREBP processing in Insig-1- and -2-deficient cells

When cellular cholesterol levels are low, ER-resident Insig proteins bind the SCAP/SREBP complex and suppress SREBP processing [25]. To examine the involvement of Insigs in piperine-mediated stimulation of SREBP processing, we performed experiments using the SRD–15 cells established by Lee *et al*. [19], which lack Insig–1 and Insig–2. In parental CHO–7 cells, the cleaved form of SREBP–2 was increased by piperine treatment and by cholesterol-depleted conditions (Fig 5C). In contrast, protein expression of the cleaved form of SREBP–2 was unaltered in SRD–15 cells cultured under sterol-depleted conditions (Fig 5D). Moreover, whereas the precursor form of SREBP–2 was barely detectable, the cleaved form of SREBP–2 gave intense signals in SRD–15 cells even under normal conditions, suggesting that SREBP processing is stimulated in SRD–15 cells. However, piperine did not increase cleaved form of SREBP–2 and may have slightly decreased it in SRD–15 cells (Fig 5D), indicating that piperine does not stimulate SREBP processing in SRD–15 cells.

### SREBPs are required for piperine-mediated stimulation of LDLR gene expression

**Fig 6:**
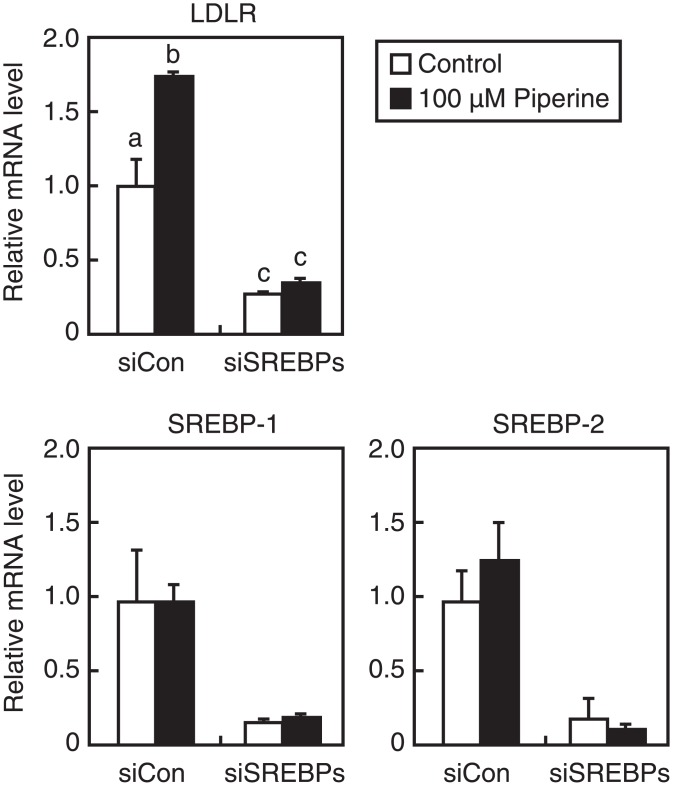
Involvement of SREBP in piperine-mediated stimulation of LDLR gene expression. HepG2 cells were transfected with 40 pmol of control siRNA (siCon) or siRNA targeting SREBP–1 and -2 (siSREBPs). Cells were then cultured in medium A for 48 h and were treated with piperine (100 μM) for 24 h. Total RNA was isolated and real-time PCR analysis was performed. Target mRNA expression levels were normalized to those of GAPDH mRNA and are presented relative to those in vehicle (DMSO)-treated siCon-transfected cells. Analyses of SREBP–1 mRNA were performed using a TaqMan probe that recognizes SREBP-1a and -1c. All data are expressed as mean ± SEM, *n* = 3. Differing letters indicate significant differences, *P < 0.05.

In final experiments, cells were transfected with siRNAs that were designed to specifically target SREBP–1 or SREBP–2 mRNA. As expected based on the fact that piperine stimulates SREBP–1 and SREBP–2 processing (Fig 4B), a single knockdown of SREBP did not affect piperine-mediated induction of LDLR gene expression (data not shown). It should be noted that SREBP-1a is the predominant isoform of SREBP–1 in HepG2 cells [26]. Cotransfection of SREBP–1 and SREBP–2 siRNAs resulted in an 80% decrease in the SREBP–1 and -2 mRNA levels and a 75% decrease in the endogenous LDLR gene expression (Fig 6). These results are consistent with previous reports showing that SREBPs regulate LDLR gene expression [24]. Moreover, piperine-mediated stimulation of LDLR gene expression was completely abolished by knockdown of SREBPs (Fig 6), confirming that piperine-mediated stimulation of LDLR gene expression is mediated by SREBPs.

## Discussion

The present data demonstrate that the alkaloid piperine stimulates LDLR gene expression and uptake of LDL particles in cultured hepatocytes. Moreover, biochemical analyses further indicated that piperine promotes the maturation of SREBPs. In agreement, the mutation of SRE of the LDLR gene promoter abolished piperine responsiveness, and the knockdown of SREBP expression abrogated the ensuing induction of LDLR gene expression.

In these experiments, we used a stable cell line that expresses a luciferase reporter gene under the control of the LDLR promoter region from −595 to +36 to identify food compounds that stimulate LDLR gene expression. Using this screening system, we found that several food compounds, including pinocembrine, 1-dodecanol, hesperetin, fisetin, piperine, and 1-tetradecanol, stimulated LDLR gene promoter activity (S1 Table). Some compounds did not stimulate LDLR promoter activity in transient reporter assays (Fig 1A) and were excluded from additional experiments. However, HEK293 cells were derived from human embryonic kidney cells and were used in transient reporter assays for their high transfection efficiency, and these compounds may stimulate LDLR promoter activity in hepatocytes. In addition, pinocembrine and fisetin did not stimulate endogenous LDLR expression but did stimulate the LDLR promoter in stable and transient reporter assays (S1 Table and Fig 1A and 1B). Although pinocembrine and fisetin stimulated LDLR promoter activity through the region examined in the present reporter assays, other upstream or downstream regions of the LDLR promoter may obscure this effect.

Several previous reports indicate that transcription, proteolytic processing, and post-translational modification are involved in the regulation of SREBPs [9]. Among these, proteolytic processing is considered a crucial regulatory step. Accordingly, under low-sterol conditions, SCAP binds to Sec23/24, leading to clustering of the SCAP/SREBP complex into common coated protein II vesicles. Subsequently, the SCAP/SREBP complex is transported from the ER to the Golgi, and SREBPs are then processed by site–1 and site–2 proteases. Given that piperine did not increase the cleavage of SREBP–2 in Insig–1 and -2-deficient SRD–15 cells (Fig 5D), piperine compromises the retention of SCAP/SREBP complexes by Insigs in the ER. Although it is unclear how piperine stimulates SREBP processing, it may directly bind Insigs or SCAP/SREBP complexes and inhibit their interactions. Recently, the crystal structure of a mycobacterial Insig homolog has been reported [27]. A homology-based structural model of human Insig–2 suggested that a central cavity of Insig–2 accommodates 25-HC and that transmembrane segments 3 and 4 of Insig–2 engage in SCAP binding. Thus, the transmembrane segments 3 and 4 of Insigs are candidates for binding with piperine. Further studies are required to determine whether piperine attenuates the binding between Insig and SCAP and whether piperine directly binds Insigs or the SCAP/SREBP complex. Alternatively, piperine may affect phosphorylation cascades that indirectly stimulate SREBP processing. It has been reported that the activation of the Akt/mTOR/S6K pathway stimulates SREBP processing [28]. On the other hand, piperine has been reported to suppress the Akt signaling pathway in osteosarcoma U2OS and breast cancer TNBC cells [29,30]. Thus, activation of Akt signaling is unlikely during piperine-mediated stimulation of SREBP processing. In agreement, treatments with the PI3K inhibitor LY294002 and the mTOR inhibitor rapamycin did not inhibit piperine-mediated stimulation of SREBP processing in HepG2 cells (data not shown).

Activated SREBPs stimulate transcription of genes involved in fatty acid and cholesterol synthesis, leading to increased lipid levels. In the present study, we demonstrated that piperine stimulated SREBP–1 and SREBP–2 processing (Fig 4B) and increased the expression of various SREBP target genes (Fig 4A). Although piperine-induced LDLR gene expression and activity may have anti-atherogenic effects, these effects may be reduced by the piperine-mediated induction of lipid synthesis genes. However, piperine treatments were reportedly beneficial in diet-induced obese mice and prevented fatty liver and corrected high-fat diet-mediated inhibition of AMPK phosphorylation [18]. Phosphorylated AMPK stimulates phosphorylation and inactivation of downstream substrates such as HMG R and ACC [31]. Thus, although piperine treatment likely stimulates the expression of lipid synthesis-related genes, simultaneous augmentation of AMPK activation may negate this effect on *de novo* lipid synthesis. We demonstrated that piperine stimulated SREBP maturation and the expression of genes related to lipid synthesis. However, piperine did not stimulate the expression of the present SREBP target genes, including PCSK9, SREBP-1c, and SREBP–2. Although the precise molecular mechanisms by which piperine stimulates specific SREBP target gene expression is unclear, piperine functions as an antagonist of the transcription factor LXRα, which reportedly stimulates SREBP-1c gene expression [17]. Thus, piperine may concomitantly stimulate SREBP activity and suppress LXRα activity, leading to no net effect of increased SREBP-1c mRNA expression. Similarly, piperine may affect the activity of transcription factors other than SREBPs, which control the expression of PCSK9 and SREBP–2, thereby abolishing the influence of SREBP on certain promoters. Further studies are essential to clarify how piperine-mediated activation of SREBPs regulates the expression of SREBP-targeted genes. PCSK9 can bind to LDLR and induce its degradation. Hence, inhibition of PCSK9 has been considered a promising drug target for atherosclerosis. Piperine-mediated activation of SREBP increased LDLR gene expression but did not affect PCSK9 expression. Thus, piperine likely has favorable anti-atherogenic activities.

Following oral administration of 170 mg/kg piperine, maximum plasma concentrations of piperine reached 11.1 μg/ml (38.8 μM) in rats [32]. In the present study, 20 μM piperine stimulated LDLR gene expression (Fig 2A) and 10 μM piperine increased SREBP processing (Fig 4C). Accordingly, treatment with a piperine derivative (GB-N) from long pepper upregulated LDLR mRNA and protein expression in rat livers [33]. Thus, induction of LDLR expression following piperine treatments likely reflects activation of SREBPs in the liver. Moreover, decreases in liver and serum cholesterol levels following piperine treatments [15–17] may reflect piperine-mediated upregulation of hepatic LDLR.

Previous studies show that piperine enhances the bioavailability of drugs, although the related mechanisms have not been characterized [13]. Nonetheless, curcumin is a well-characterized constituent of the common food spice turmeric (*Curcuma longa*), and its bioavailability is reportedly enhanced by concomitant administration of piperine [32]. Moreover, a recent report indicated that curcumin downregulates PCSK9 gene expression, thereby stimulating cell-surface LDLR protein expression and promoting LDL uptake into HepG2 cells [34]. These results suggest that concomitant administration of piperine and curcumin stimulates LDLR activity via multiple pathways, including (1) piperine-meditated stimulation of LDLR gene expression, (2) curcumin-meditated suppression of PCSK9 gene expression, and (3) piperine-mediated enhancement of the bioavailability of curcumin. However, further studies are required to confirm these effects and to improve therapeutic options for atherosclerosis.

In summary, the present data demonstrate that piperine stimulates SREBP activity. In addition, we show that piperine-mediated activation of SREBP increases LDLR gene expression and consequent LDL uptake into HepG2 cells. Essential future studies will determine whether dietary piperine affects hepatic LDLR expression *in vivo*.

## Supporting Information

S1 Table Effects of the test compounds (100 μM) on LDLR promoter activity. Luciferase assays were performed in triplicate using Huh7/LDLR-Luc cells as described in the Materials and Methods section.
(EPS)
